# Supplementary figures and images for: Identification of triciribine as a novel myeloid cell differentiation inducer
Source: PLoS One. 2024 May 14;19(5):e0303428. doi: 10.1371/journal.pone.0303428 (PMC11093380; doi:10.1371/journal.pone.0303428)

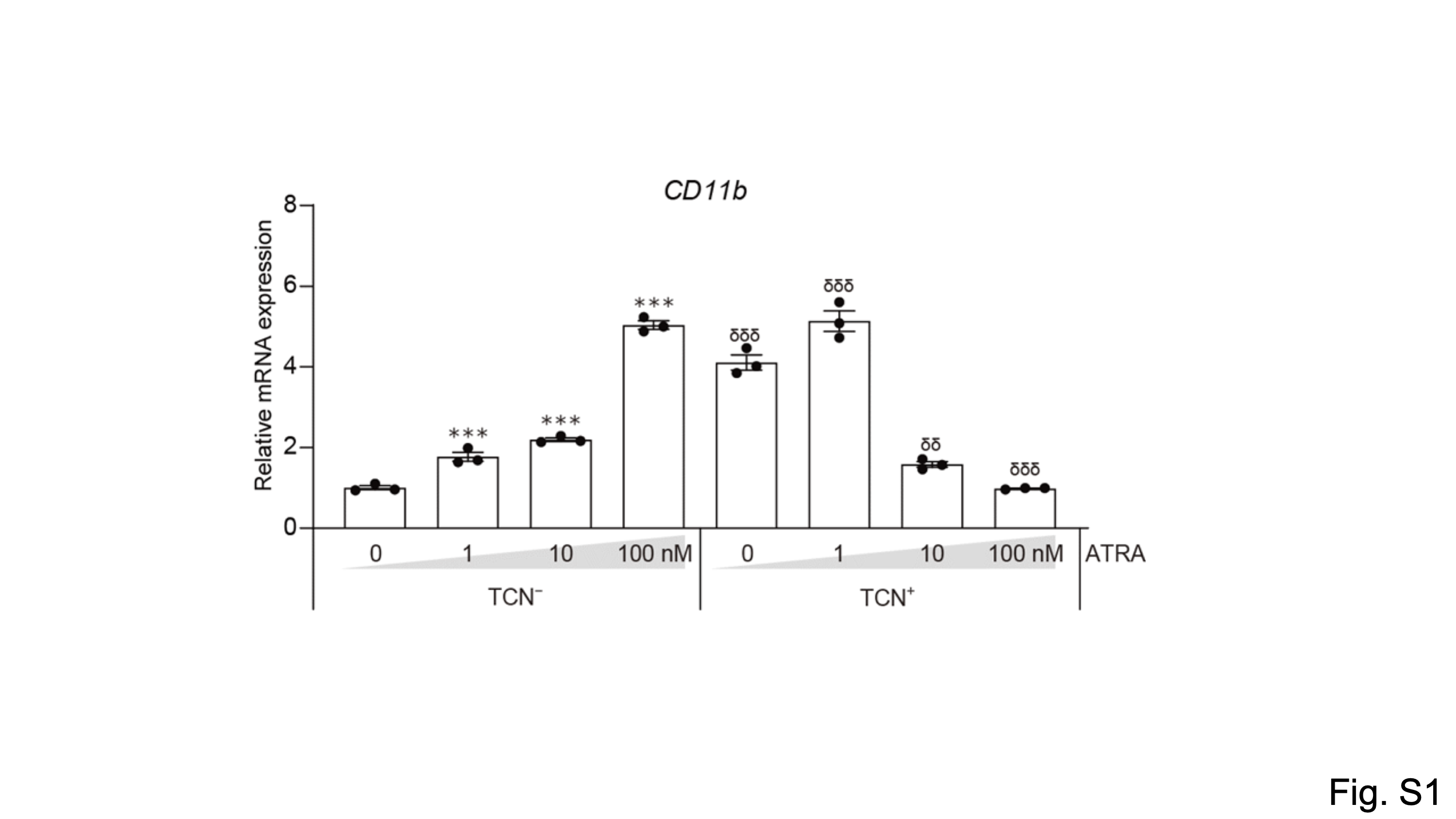

Supplement: S1 Fig — Relative mRNA expression was calculated from the copy number of the target gene adjusted by GAPDH, in each TCN-treated sample (10 μM), or ATRA-treated sample (1, 10, 100 nM), which was then divided by the control sample values. The data shown were obtained from three independent PCR amplifications Statistical significance was determined by two-way ANOVA followed by Tukey’s multiple comparisons test (*** p<0.001 vs. ctrl, δδ p<0.01, δδδ p<0.001 vs. ATRA). (TIFF) [file pone.0303428.s001.tiff]

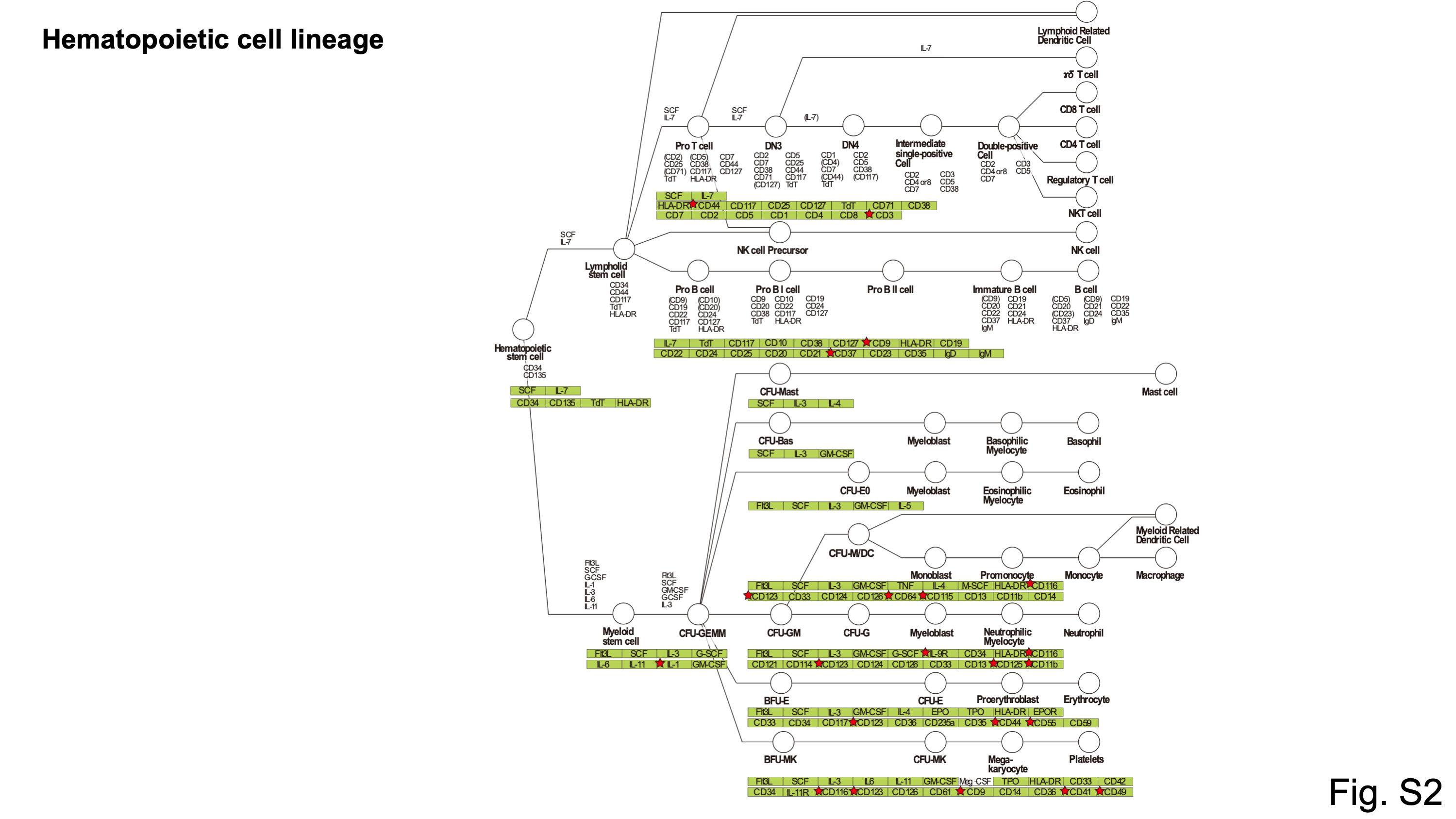

Supplement: S2 Fig — Red stars are genes with expressions induced more than 3-fold by TCN compared with controls. (TIFF) [file pone.0303428.s002.tiff]

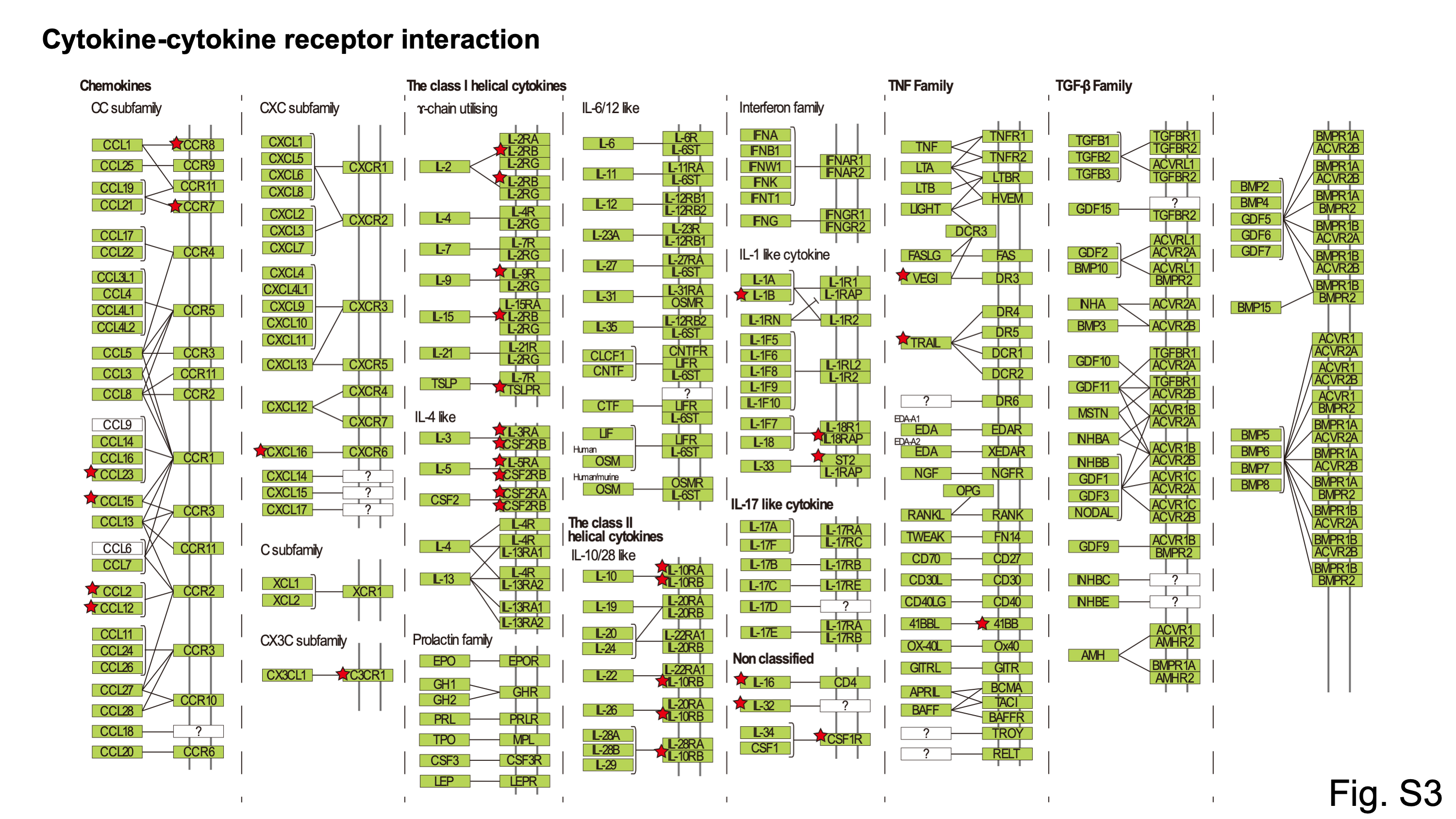

Supplement: S3 Fig — Red stars are genes with expressions induced more than 3-fold by TCN compared with controls. (TIFF) [file pone.0303428.s003.tiff]
