# Supplementary material for: Identification of triciribine as a novel myeloid cell differentiation inducer
Source: PLoS One. 2024 May 14;19(5):e0303428. doi: 10.1371/journal.pone.0303428 (PMC11093380; doi:10.1371/journal.pone.0303428)
Supplement: S1 Raw images — (PDF) [file pone.0303428.s004.pdf]

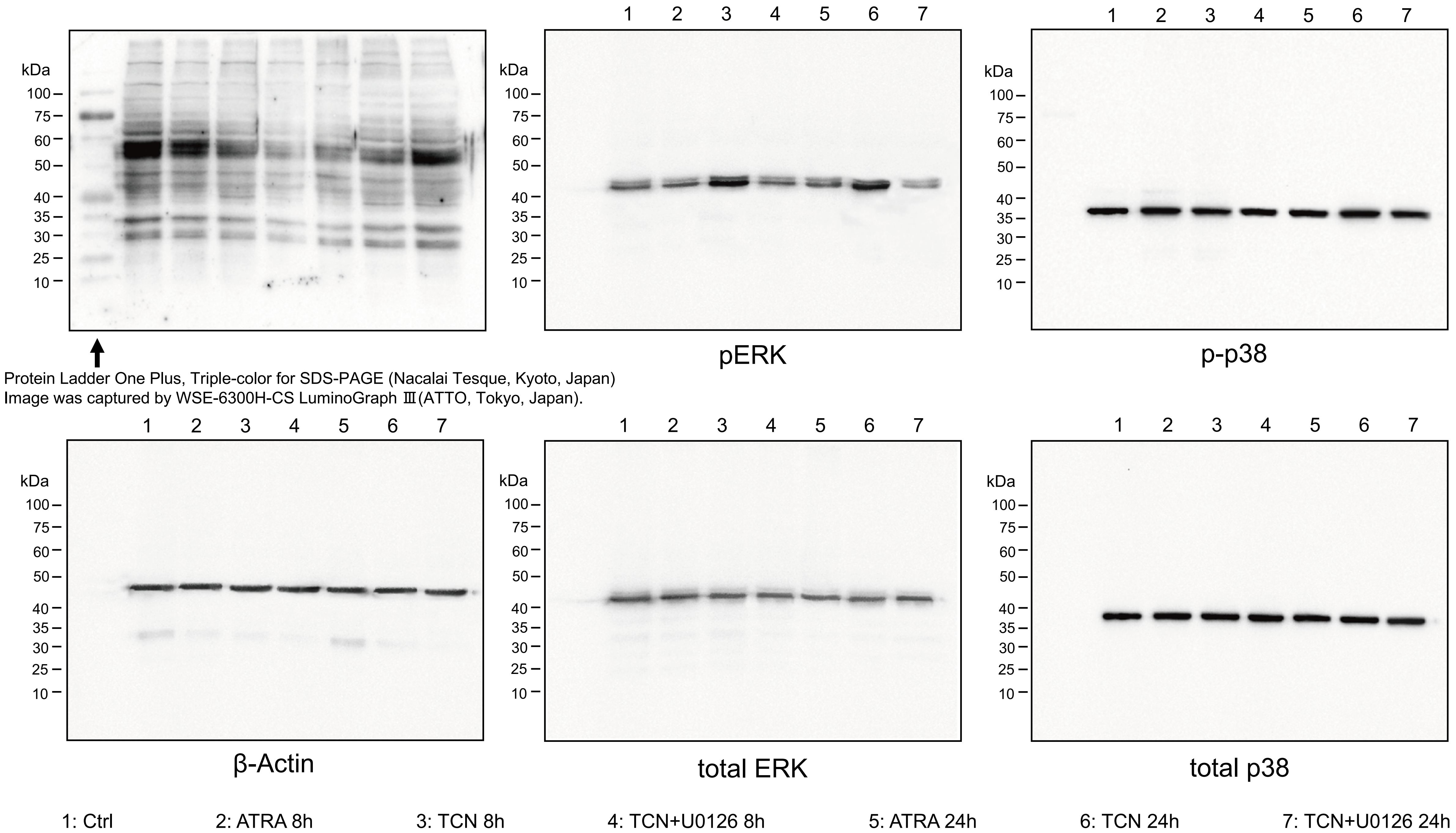
kDa
100
75
60
50
40
35
30
25
10
1 2 3 4 5 6 7
pERK
p-p38
Protein Ladder One Plus, Triple-color for SDS-PAGE (Nacalai Tesque, Kyoto, Japan)
Image was captured by WSE-6300H-CS LuminoGraph Ⅲ(ATTO, Tokyo, Japan).
β-Actin
total ERK
total p38
1: Ctrl
2: ATRA 8h
3: TCN 8h
4: TCN+U0126 8h
5: ATRA 24h
6: TCN 24h
7: TCN+U0126 24h
