# Supplementary material for: Identification of triciribine as a novel myeloid cell differentiation inducer
Source: PLoS One. 2024 May 14;19(5):e0303428. doi: 10.1371/journal.pone.0303428 (PMC11093380; doi:10.1371/journal.pone.0303428)
Supplement: Attachment — Submitted filename: Point by point reply to reviewers0227.doc [file pone.0303428.s008.doc]

**Reviewer #1: The authors have presented an interesting and clinically important study. However, the following observations have been made considering the study aims to prove that TCN can be used as a clinical alternative to ATRA in the treatment of AML:
The study only compares the effects of ATRA and other kinase inhibitors on NBT positive cells and the expression of CD11b gene.
The study should compare the effect of ATRA to TCN on:
1. The morphology of NB4 cells
2. The induction of myelomonocytic and other differentiation markers in markers in NB4 cells, especially CD11b, CD11c, CD14 and MPO, considering TCN only effectively induces CD11b and CD11c at high concentrations
3. The signaling pathways in NB4 cells
These comparisons would make for a more robust study on the role of TCN in myeloid differentiation and as an effective alternative to ATRA.**

Thank you very much for these constructive comments. To follow this, we compared the results of the effect of ATRA on the 1. morphology, and 2. differentiation markers, not only in NB4 cells, but in this revised version, we employed AML (M2) derived (13) cell line HL-60 cells. These results are incorporated in the revised manuscript as follows,

Nuclear/cytoplasmic (N/C) ratio was significantly decreased, and myelomonocytic markers (*CD11b* and *CD11c*) were potently induced by TCN in both NB4 and acute myeloid leukemia (AML) M2 derived HL-60 cells. (line 25)

To prove the efficiency of TCN in inducing cell differentiation in other AML cell types other than NB4, we examined an AML (M2) derived (13) cell line HL-60. As shown in Fig. 3A, 72 hours after the addition of TCN, the cytoplasm of NB4 cells was enlarged with slight budding of the plasma membrane region, which was morphologically similar to the effect of 100 nM of ATRA, at the concentration we observed similar nuclear/cytoplasmic (N/C) ratio to 10 µM of TCN (Fig 3B). (line 180)

Next, we evaluated N/C ratio, which was analyzed statistically and presented as a violin plot. In the absence of TCN, the mean N/C ratio was 70%, which was significantly decreased to 60% in the presence of TCN, which was similar to the effect of the addition of 100 nM ATRA (16) (Fig. 3B). In HL-60 cells, the cytoplasm was enlarged like which was seen in NB4 cells (Fig. 3C), however, the effect of TCN was even more efficient than 100 nM of ATRA (Fig. 3D). (line 188)

Similar effect was also observed in HL-60 cells. We next examined the effect of ATRA on *CD11b, CD11c, CD14*, *and myeloperoxidase (MPO)* genes. Real-time PCR analyses demonstrated that ATRA potently increased the expressions of myelomonocytic markers *CD11b*, *CD11c,* whereas *MPO* expression was decreased, in a dose-dependent manner in both NB4 and HL-60 cells (Fig. 4 E-H), which was similar to the effect of TCN effect we observed (Fig. 4 A-D). In addition, CD14 expression is induced by ATRA in NB4 cells but not in HL-60 cells. (line 211)

Moreover, we examined 3. signaling affected by ATRA and TCN in NB4 cells and the results were incorporated as follows,

We further examined that whether ATRA may affect phosphorylation of ERK and p38, and found that there was no obvious effect compared to TCN, suggesting that ATRA induced differentiation is different from TCN effect. (line 30)

We also examined that whether ATRA may affect phosphorylation of ERK and p38, and found that there was no obvious effect compared to TCN, suggesting that mechanisms of ATRA induced differentiation is different from TCN effect. In addition, TCN induced phosphorylation of ERK was completely abrogated by the addition of MEK inhibitor U0126, suggesting that the involvement of MEK/ERK pathway. We further examined the combination effect of TCN and ATRA for the induction of CD11b expression, and found that there was no enhancement effect (data not shown), may also suggest the difference of the mechanisms of these agents. To verify whether the ERK pathway has a role in TCN induced differentiation, we confirmed that the induction of CD11b expression was efficiently reduced by the addition of U0126 (Fig. 5B). Collectively, activation of the MEK/ERK pathway is involved in TCN-induced differentiation and this is distinctly different from ATRA induced differentiation.

(line 241)

Additionally, we examined the combination effect of TCN with ATRA in NB4 cells, because other reviewer requested, and found that there was no effect for CD11b induction (as below). This may also suggest that the mechanisms of the effect of TCN is different from ATRA.

I appreciate very much for these constructive comments. Following these comments, I feel that quality of our paper improved greatly. Thank you so much.

**Reviewer #2: In this manuscript, Suzuki and colleagues propose the use of Triciribine (TCN), an Akt inhibitor, to potentially improve the treatment of AML because it induces cell differentiation otherwise only previously shown to reduce the growth and survival of cancer cells. TCN has already been tested in a phase I clinical trial for the treatment of advanced hematological malignancies and has been shown to be well tolerated by patients. They used the NB4 cell line to test its cell differentiation efficiency and related pathways activated by the drug in in vitro assays. Although the results were innovative and the experiments were well designed, I have few comments regarding the conceptualization:
1) The authors use the following phrase “because the narrow application and tolerance development of ATRA need to be improved, we searched for another efficient myeloid differentiation inducer” as a rationale for improving the treatment of patients with AML/APL. In this sense, in addition to the NB4 cell line (sensitive to ATRA), they should have tested the efficiency of TNC in cell lines resistant to ATRA. Or prove its efficiency in inducing cell differentiation in other AML cell types other than NB4 (an APL cell line).**

Thank you very much for pointing this out. To follow this, in this revised version, we employed AML (M2) derived (13) cell line HL-60 cells, which is other AML cell types than NB4. These results are incorporated in the revised manuscript as follows,

To prove the efficiency of TCN in inducing cell differentiation in other AML cell types other than NB4, we examined an AML (M2) derived (13) cell line HL-60. (line 180)

In HL-60 cells, the cytoplasm was enlarged like which was seen in NB4 cells (Fig. 3C), however, the effect of TCN was even more efficient than 100 nM of ATRA (Fig. 3D). (line 191)

Similar effect was also observed in HL-60 cells. (line 212)

These results suggest that a high concentration of TCN efficiently induces myelomonocytic markers (*CD11b* and *CD11c*) in NB4 and HL-60 cells. (line 219)

**2) If they only have access to the NB4 cell line. Why not test drug combinations? Is TNC+ATRA superior in inducing cell differentiation or cell death? It would make more sense, since ATRA (low dose) is still superior to TCN in inducing cell differentiation in APL cells.**

In this revised version, we examined the effect of TCN not only to NB4 cells but AML M2 derived HL-60 cells, which is other AML cell types than NB4. As a result, we found that TCN is also effective in HL-60 cells as described above.

Moreover, to follow this comment, we examined the combination effect of TCN with ATRA in NB4 cells, and found that there was no effect for CD11b induction (as below). This may probably from the mechanisms for the induction of differentiation by TCN is different from ATRA, in terms of ERK phosphorylation.

These are incorporated in Results, and Discussion, as follows:

We further examined the combination effect of TCN and ATRA for the induction of CD11b expression, and found that there was no enhancement effect (data not shown), may also suggest the difference of the mechanisms of these agents. (line 246)

In the current study, we demonstrated that the signaling effect to ERK, are completely different between TCN and ATRA in NB4 cells. This fact may suggest that synergistic interactions cannot be expected for these two agents. In fact, there was no obvious positive combinational effect for CD11b expression in NB4 cells (data not shown). (line 320)

However, as pointed out, it is very interesting to see the combination effect of ATRA and TCN to other cell lines than APL. It would be grateful if you allow us to move forward this time and would like to examine this as a future project.

**3) Line 256: “L) total RNAs from NB4 cells cultured in the presence or absence of ATRA and/or 6AF”. Did the authors want to write TCN instead of 6AF?**

Absolutely, and we sincerely apologize for this mistake. We intended to write TCN, instead of 6AF. Thank you very much for pointing this out.

By following these comments, we feel our paper has improved so much. We really appreciate this.
