# Supplementary material for: Identification of triciribine as a novel myeloid cell differentiation inducer
Source: PLoS One. 2024 May 14;19(5):e0303428. doi: 10.1371/journal.pone.0303428 (PMC11093380; doi:10.1371/journal.pone.0303428)
Supplement: Attachment — Submitted filename: Point by point reply to reviewers0412.doc [file pone.0303428.s009.doc]

**Reviewer #2: The authors provided significant improvements to the manuscript. However, I recommend some edits and another experiment to support the data that has been added to this manuscript and increase the relevance of this study.**

**Abstract: It is missing a conclusion showing the relevance of this study to support a future clinical perspective.**

Thank you very much for this constructive comment. To follow this, we added following sentence in abstract (line 38),

Collectively, we identified TCN as a novel myeloid cell differentiation inducer, and trials of TCN for APL and non-APL leukemia are worthy of exploration in the future.

**Line 217: Please revise the sentence “which was similar to the effect of TCN effect we observed” and replace it by “which was similar to the TCN effect we observed.”**

Thank you for pointing this out. We corrected as follows (line 218),

which was similar to the TCN effect we observed (Fig. 4A-D).

**Lines 217-218: Please correct the sentence “In addition, CD14 expression is induced by ATRA in NB4 cells but not in HL-60 cells.” Figure 4G shows that ATRA induces increased CD14 expression in both cell lineages (NB4 and HL-60).**

We apologize for this mistake. We corrected as follows (line 218),

In addition, CD14 expression is induced by ATRA in both lineages.

**Do you consider the involvement of the MEK/ERK signaling pathway mediating the cell differentiation process of AML cell lines other than NB4 cells (in this case HL-60)? ATRA is the best inducer of cell differentiation for NB4 cells, but TCN significantly reduced the N/C ratio in HL-60 cells when compared to ATRA (Fig. 3D). Although in the discussion section the authors commented about the relevance of other agents in inducing cell differentiation through the activation of the MEK/ERK pathway in HL-60 cells, I would recommend the authors to perform the same western blot assay (Fig. 5) for HL-60 cells while trying to highlight the novelty of using TCN as a relevant inducer of cell differentiation for other AML types. It would increase the relevance for future research and a potential clinical use of TCN for AML not APL.**

Yes, we think MEK/ERK signaling pathway mediates the cell differentiation process by TCN in HL-60 cells as well. However, we don’t think it is good to present HL-60 Western blot in parallel to NB4 (Fig. 5A). We currently found that when combined with ATRA, TCN effect for gene expression is different from NB4 (Fig. S1) to HL-60 cells. If we present the Western blot for TCN effect using HL-60 cells, it is better to present TCN+ATRA combination effect for Western blot, gene expressions analysis, and even morphological study in both cell lines. In that case, it may be better to add microarray analysis using HL-60 cells, to see the difference between NB4 cells.

Originally, the main focus of this paper is about the identification of TCN through the screening of kinase inhibitor library, and clarifying the mechanisms of TCN effect by microarray, by using NB4 APL cells. To follow reviewers’ comments at 1^st^ revision, we compared the effect of TCN and ATRA, revealed the sufficient effect of TCN on non-APL cell line, HL-60 AML cells. The query about the mechanism of TCN for HL-60 AML cells is raised from this 2^nd^ revision, but to answer this, we think that this needs to be thoroughly investigated, which will surely take some time. Indeed, we are now investigating this TCN effect, combined with ATRA, in many AML cells. We started to examine for signaling and gene expressions to reveal the mechanisms, because we also think it is important and to increase the relevance of this series of research.

It would be grateful if you allow as to move forward at this time, and clarify this as a next project.

**Lines 246-248: “We further examined the combination effect of TCN and ATRA for the induction of CD11b expression and found that there was no enhancement effect (data not shown), may also suggest the difference of the mechanisms of these agents.” Why not show the data as supporting information? It is important to show that these drugs with pro-differentiation effects together do not increase cell differentiation in the cell lines tested as an available data. After adding the data, please remove “data not shown” from the discussion section (line 324).**

We agree with this suggestion. We added this data in Fig S1.

Following these comments, we feel the improvement of our paper. We appreciate very much for these comments.
